# Additions to Chaetothyriaceae (Chaetothyriales): *Longihyalospora* gen. nov. and *Ceramothyrium longivolcaniforme*, a new host record from decaying leaves of *Ficus ampelas*

**DOI:** 10.3897/mycokeys.61.47056

**Published:** 2019-12-16

**Authors:** Danushka S. Tennakoon, Kasun M. Thambugala, Rajesh Jeewon, Sinang Hongsanan, Chang-Hsin Kuo, Kevin D. Hyde

**Affiliations:** 1 Department of Plant Medicine, National Chiayi University, 300 Syuefu Road, Chiayi City 60004, Taiwan Mae Fah Luang University Chiang Rai Thailand; 2 Center of Excellence in Fungal Research, Mae Fah Luang University, Chiang Rai, 57100, Thailand National Chiayi University Chiayi Taiwan; 3 Key Laboratory for Plant Biodiversity and Biogeography of East Asia (KLPB), Kunming Institute of Botany, Chinese Academy of Science, Kunming 650201, Yunnan, China Kunming Institute of Botany, Chinese Academy of Science Kunming China; 4 Industrial Science and Management (International Program), Faculty of Science and Technology, Thammasat University (Rangsit Center), Klong Luang, Pathumthani 12121, Thailand Thammasat University Pathumthani Thailand; 5 Department of Health Sciences, Faculty of Science, University of Mauritius, Reduit, Mauritius University of Mauritius Moka Mauritius; 6 Shenzhen Key Laboratory of Microbial Genetic Engineering, College of Life Sciences and Oceanography, Shenzhen University, Shenzhen 518000, China Shenzhen University Shenzhen China

**Keywords:** Moraceae, multi-gene phylogeny, mycelium pellicle, sooty mould, taxonomy

## Abstract

A novel ascomycete genus, *Longihyalospora*, occurring on leaf litter of *Ficus ampelas* in Dahu Forest Area in Chiayi, Taiwan is described and illustrated. *Longihyalospora* is characterized by dark mycelium covering the upper leaf surface, elongate mycelial pellicle with ring of setae, pale brown to brown peridium, broadly obovoid, short pedicellate asci and hyaline, fusiform, elongated (tapering ends) and multi-septate ascospores with a thin mucilaginous sheath. Phylogenetic analyses of combined ITS, LSU and SSU sequence data revealed *Longihyalospora* as a distinct genus within the Chaetothyriaceae with high bootstrap support. Moreover, based on morphological similarities, *Chaetothyrium vermisporum* transferred to the new genus. In addition, *Ceramothyrium longivolcaniforme* is reported for the first time on *Ficus ampelas*. Newly added species are compared with other similar species and comprehensive descriptions and micrographs are provided.

## Introduction

The family Chaetothyriaceae was established by Hansford (1946) with the generic type *Chaetothyrium* Speg., and the members of this family are characterized by a loose network of dark mycelium over the substrate, ascomata produced beneath a mycelial pellicle, and forming beneath an external hyphal mat with or without setae (Batista and Ciferri 1962; von Arx and Müller 1975; Hughes 1976; Pereira et al. 2009; Chomnunti et al. 2012; Tian et al. 2014; Zeng et al. 2016). Due to some morphological similarities (i.e. bitunicate asci), Eriksson (1982) referred this family to the order Dothideales in Dothideomycetes, but subsequently, taxonomic studies have established its placement in Eurotiomycetes with support of molecular data (Chomnunti et al. 2012, 2014; Tian et al. 2014; Crous et al. 2015; Maharachchikumbura et al. 2018; Yang et al. 2018). Currently, 16 genera are accepted in Chaetothyriaceae, viz. *Actinocymbe* Höhn., *Aphanophora* Réblová & Unter., *Beelia* F. Stevens & R.W. Ryan, *Camptophora* Réblová & Unter., *Ceramothyrium* Bat. & H. Maia, *Ceratocarpia* Rolland, *Chaetothyriomyces* Pereira-Carvalho et al., *Chaetothyrium* Speg., *Cyphellophoriella* Crous & A.J. Sm., *Euceramia* Bat. & Cif., *Microcallis* Syd., *Phaeosaccardinula* P. Henn., *Stanhughesia* Constant., *Treubiomyces* Höhn., *Vonarxia* Bat. and *Yatesula* Syd. & P. Syd. (Wijayawardene et al. 2018).

During our survey of the taxonomy and diversity of leaf litter microfungi, two interesting fungal species were collected from Dahu forest, Chiayi in Taiwan. Morphological and multi-gene phylogenetic analyses were performed to establish their taxonomic placement.

## Materials and methods

### Sample collection, morphological studies and isolation

Decaying leaf litter samples of *Ficus ampelas* Burm.f. were collected from Dahu forest area in Chiayi, Taiwan and brought to the laboratory in plastic bags. The samples were incubated in plastic boxes at 25–30 °C for 3 days and examined following the methods described by Tian et al. (2014). Morphological observations were made using an Axioskop 2 Plus compound microscope and images were taken with an Axioskop 2 Plus compound microscope equipped with a Canon Axiocam 506 Color digital camera. Permanent slides were prepared by mounting fungal material in lactoglycerol and sealed by applying nail-polish around the margins of cover slips. All measurements were made with ZEN2 (blue edition) and images used for figures were processed with Adobe Photoshop CS3 Extended version 10.0 software (Adobe Systems, USA).

Isolates (for *Ceramothyrium longivolcaniforme* Zeng, T.C. Wen & K.D. Hyde) were obtained from single ascospores following the methods described in Chomnunti et al. (2014). Germinated ascospores were transferred to potato dextrose agar (PDA) and incubated at 25 °C in normal light. Subsequent sub culturing was done carefully to ensure no contaminants are used to generate DNA sequence data. Culture characteristics were observed after two weeks. Type specimens were deposited in the Mae Fah Luang University Herbarium (MFLU) and living cultures were deposited in Mae Fah Luang University Culture Collection (MFLUCC). Faces of Fungi and Index Fungorum numbers were provided as in Jayasiri et al. (2015) and Index Fungorum (2019).

### DNA extraction and PCR amplification

Fresh mycelia were scraped (for *Ceramothyrium longivolcaniforme*) using a sterile scalpel from pure cultures growing on PDA medium at 25 °C and kept in a 1.5 ml micro-centrifuge tube and used as starting material for DNA extraction. When fungi failed to germinate in a culture medium, DNA was extracted directly from ascomycete fruiting bodies (for *Longihyalospora ampeli*) by following a modified protocol of Zeng et al. (2018) protocol: 15–20 fruiting bodies (> 500 µm diam., 10 fruiting bodies) were removed from the host substrate using a sterilized needle and transferred to a drop of sterile water, placed in a sterile Eppendorf tube (1.5 mL) under aseptic conditions.

The genomic DNA was extracted using a DNA extraction kit (E.Z.N.A Fungal DNA Mini Kit, D3390-02, Omega Bio-Tek) following the manufacturer’s protocol. The DNA product was kept at 4 °C for DNA amplification and maintained at -20 °C for long-term storage. DNA was amplified by Polymerase Chain Reaction (PCR) for three genes, the large subunit (28S, LSU), small subunit (18S, SSU) and internal transcribed spacers (ITS1-5.8S-ITS2). The LSU gene was amplified by using the primers LR0R and LR5 (Vilgalys and Hester 1990; Rehner and Samuels 1994); SSU gene was amplified using the primers NS1 and NS4 (White et al. 1990); nuclear ITS was amplified by using the primers ITS5 and ITS4 (White et al. 1990). The amplification reactions were performed in 25µl of total reaction that contained 9.5 µl of sterilized water, 12.5 µl of 2×Power Taq PCR MasterMix (Tri-I Biotech, Taipei, Taiwan), 1 μl of each forward and reverse primers and 1 μl of DNA template. PCR thermal cycle program for ITS, LSU and SSU were as detailed by Tian et al. (2016). The PCR products were analyzed by 1.5% agarose gels containing the Safeview DNA stain (GeneMark, Taipei, Taiwan) to confirm the expected molecular weight of a single amplification product. PCR products were purified and sequenced with primers mentioned above by Tri-I Biotech, Taipei, Taiwan. Nucleotide sequences were deposited in GenBank (Table 1).

**Table 1.** GenBank and culture collection accession numbers of species included in the present phylogenetic study. The newly generated sequences are shown in bold.

Species	Strain/Voucher no.	GenBank accession no.		
		ITS	LSU	SSU
*Aphanophora eugeniae*	CBS 124105	FJ839617	FJ839652	–
*Brycekendrickomyces acaciae*	CBS 124104	MH863350	MH874874	–
*Camptophora hylomeconis*	IFRDCC 2661	MF285228	MF285230	–
*C. hylomeconis*	CBS 113311	EU035415	–	KC455295
*Capronia fungicola*	CBS 614.96	KY484990	FJ358224	FJ225722
*C. mansonii*	CBS 101.67	AF050247	MH870591	AF346422
*Ceramothyrium aquaticum*	LC306299	LC360299	LC360296	–
*C. carniolicum*	AFTOL-ID 1063	–	EF413628	EF413627
*C. carniolicum*	CBS 175.95	KC978733	KC455251	KC455294
*C. exiguum*	LC306297	LC360297	LC360295	–
*C. ficus*	MFLUCC 15-0228	KT588601	KT588599	–
*C. ficus*	MFLUCC 15-0229	KT588602	KT588600	–
*C. longivolcaniforme*	MFLU 16-1306	KP324929	KP324931	–
***C. longivolcaniforme***	**MFLUCC 19-0252**	**MN219715**	**MN238770**	**MN238773**
*C. melastoma*	CPC 19837	KC005771	KC005793	–
*C. menglunense*	MFLU 16-1874	KX524148	KX524146	–
*C. phuquocense*	LC306298	LC360298	LC360294	–
*C. podocarpi*	CPC 19826	KC005773	KC005795	–
*C. thailandicum*	MFLUCC 10-0008	KP324928	HQ895835	–
*C. thailandicum*	MFLU 13-0632	HQ895838	KP324930	–
*Chaetothyrium agathis*	MFLUCC 12-0113	KP744437	KP744480	–
*C. brischoficola*	MFLUCC 10-0012	HQ895839	HQ895836	–
*Cladophialophora minourae*	CBS 556.83	AY251087	FJ358235	FJ225734
*C. emmonsii*	CBS 640.96	KX822192	KC809995	KX822192
*Cyphellophoriella pruni*	CPC 25120	KR611878	–	–
*Leptoxyphium fumago*	CBS 123.26	MH854862	GU214430	GU214535
*L. madagascariense*	CBS 124766	MH863407	GQ303308	–
***Longihyalospora ampeli***	**MFLU 19-0824**	**MN219716**	**MN238771**	**MN238774**
***L. ampeli***	**MFLU 19-0825**	**MN219717**	**MN238772**	**MN238775**
*Knufia cryptophialidica*	DAOM 216555	–	JN040500	EF137364
*K. cryptophialidica*	DAOM 216553	JN040504	–	EF137363
*K. perforans*	CBS 885.95	MH862564	MH874191	–
*K. perforans*	CBS 726.95	KC978746	KC978741	KC978739
*Minimelanolocus asiaticus*	MFLUCC 15-0237	KR215604	KR215610	KR215615
*M. melanicus*	MFLUCC 15-0415	KR215608	KR215613	KR215618
*Phaeosaccardinula dendrocalami*	IFRDCC 2663	KF667243	KF667246	–
*P. dendrocalami*	IFRDCC 2649	KF667242	KF667245	–
*P. ficus*	MFLUCC 10-0009	HQ895840	HQ895837	–
*P. multiseptata*	IFRDCC 2639	KF667241	KF667244	–
*Trichomerium deniqulatum*	MFLUCC 10-0884	JX313654	JX313660	–
*T. follicola*	MFLUCC 10-0058	JX313653	JX313659	–
*T. gleosporum*	MFLUCC 10-0087	JX313656	JX313662	–
*Vonarxia vagans*	CBS 123533	FJ839636	FJ839672	KC455310
*V. vagans*	CPC 15152	FJ839637	FJ839673	–

### Phylogenetic analysis

Phylogenetic analyses were performed based on a combined ITS, LSU and SSU DNA sequence data. Newly generated sequences were subjected to a standard BLAST search of GenBank to aid in phylogenetic taxon sampling. Other sequences used in the analyses (Table 1) were obtained from GenBank based on recently published data (Zeng et al. 2016; Maharachchikumbura et al. 2018; Yang et al. 2018). The multiple alignments were made with MAFFT v. 7 at the web server (http://mafft.cbrc.jp/alignment/server), using default settings (Katoh and Standley 2013). The alignment was refined manually with BioEdit v. 7.0.5.2 (Hall 1999) where necessary. The tree topologies obtained from a single gene sequence data were compared prior to the combined gene analysis for checking the incongruence in overall topology of the phylogenetic tree.

Maximum likelihood trees were generated using the RAxML-HPC2 on XSEDE (8.2.8) (Stamatakis et al. 2008; Stamatakis 2014) in the CIPRES Science Gateway platform (Miller et al. 2010) using GTRGAMMA model with 1,000 bootstrap replicates. Maximum parsimony analysis (MP) was performed in PAUP v. 4.0b10 (Swofford 2002), with the heuristic search option and 1,000 random replicates. Maxtrees was set to 1,000 and branches of zero length were collapsed and all multiple parsimonious trees were saved. Descriptive tree statistics for parsimony (Tree Length [TL], Consistency Index [CI], Retention Index [RI], Relative Consistency Index [RC] and Homoplasy Index [HI] were calculated.

A Bayesian analysis (GTR+I+G model) was conducted with MrBayes v. 3.1.2 (Huelsenbeck and Ronqvist 2001) to evaluate posterior probabilities (PP) (Rannala and Yang 1996; Zhaxybayeva and Gogarten 2002) by Markov Chain Monte Carlo sampling (BMCMC). Six simultaneous Markov chains were run for 1,000,000 generations and trees were sampled every 100^th^ generation, thus 10,000 trees were obtained. The suitable burn-in phases were determined by inspecting likelihoods and parameters in Tracer version 1.6 (Rambaut et al. 2014). Based on the tracer analysis, the first 1,000 trees representing 10% were discarded as the burn-in phase in the analysis. The remaining trees were used to calculate posterior probabilities in the majority rule consensus tree (critical value for the topological convergence diagnostic set to 0.01). Phylograms were visualized with FigTree v1.4.0 (Rambaut 2012) and annotated in Microsoft Power Point (2010). The final alignment and trees were deposited in TreeBASE, submission ID: 24826.

## Results

### Phylogenetic analysis

**Figure 1.**
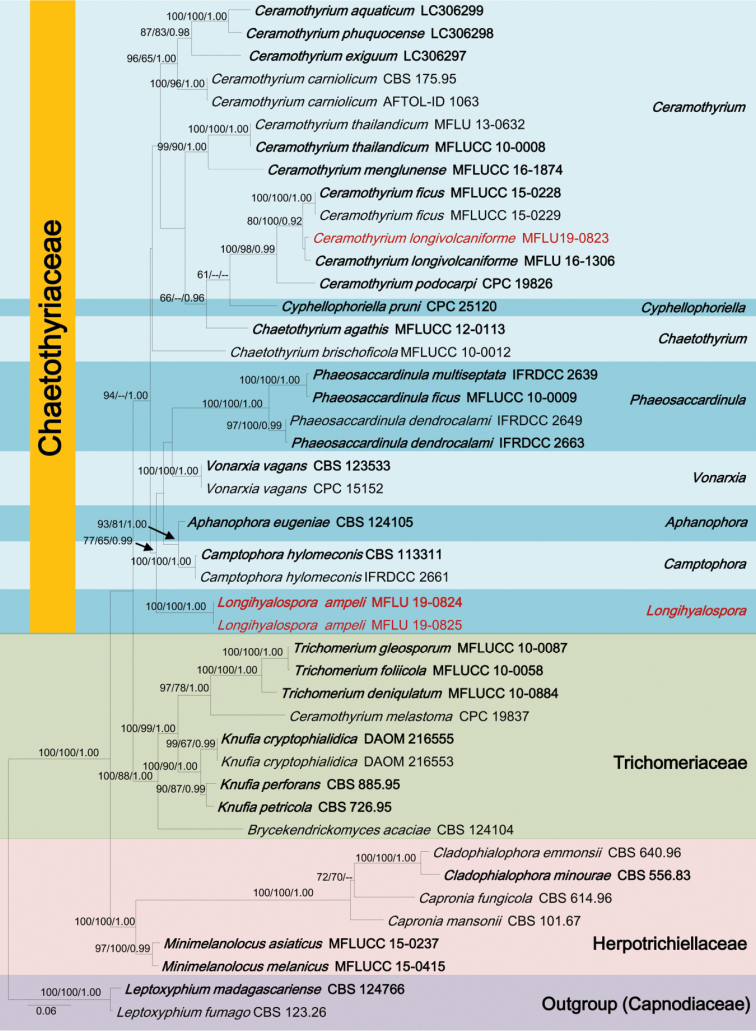
RAxML tree based on a combined dataset of ITS, LSU and SSU partial sequences of 45 taxa. Bootstrap support values for maximum likelihood (ML and, maximum parsimony (MP) values higher than 60 % and Bayesian posterior probabilities (BYPP) greater than 0.90 are given above each branch respectively. The new isolates are in red. Ex-type strains are in bold. The tree is rooted by *Leptoxyphium fumago* (CBS 123.26) and *L. madagascariense* (CBS 124766).

The combined dataset of ITS, LSU and SSU sequences comprised 2531 characters, of which 1492 characters are constant, 801 characters are parsimony-informative, while 238 variable characters are parsimony-uninformative in the maximum parsimony (MP) analysis (TL = 3011, CI = 0.515, RI = 0.698, RC = 0.360, HI = 0.485). LSU contains 900 total characters (constant = 645, informative = 217, uninformative = 38), ITS contains 759 total characters (constant = 332, informative = 364, uninformative = 63) and SSU contains 872 characters (constant = 515, informative = 220, uninformative = 137). The RAxML analysis of the combined dataset yielded a best scoring tree (Figure 1) with a final ML optimization likelihood value of -17222.496803. The matrix had 1040 distinct alignment patterns, with 37.84 % of undetermined characters or gaps. All analyses (ML, MP and BYPP) gave similar results and in agreement with previous studies based on multi-gene analyses (Zeng et al. 2016; Maharachchikumbura et al. 2018).

The phylogeny recovered herein also agrees with previously established ones in that *Ceramothyrium* is within the Chaetothyriales (Zeng et al. 2016; Maharachchikumbura et al. 2018; Yang et al. 2018). Our new collection (MFLUCC19-0252) grouped in a well-supported clade (80% ML, 100% MP and 0.92 BYPP) with other *Ceramothyrium* species (Figure 1). In particular, it shows a close affinity to *Ceramothyrium longivolcaniforme* (holotype, MFLU16-1306). MFLU 19-0824 and MFLU 19-0825 constitute in a strongly supported subclade and is phylogenetically distinct from other genera in family (77% ML, 65% MP, 0.99 BYPP) (Figure 1).

### Taxonomy

#### Ceramothyrium longivolcaniforme

Taxon classificationAnimaliaChaetothyrialesChaetothyriaceae

X.Y. Zeng, T.C. Wen & K.D. Hyde, Phytotaxa 267(1): 54 (2016)

E34C0D23-711F-5F35-8434-789F952FE966

Fungorum Number: IF 811216

Facesoffungi number: FoF0047

Figure 2

##### Description.

*Epiphytic* on decaying leaves of *Ficus ampelas* Burm.f. Covering the upper leaf surface with dark mycelium without penetrating host tissues. *Mycelial pellicle* elongate, subiculum-like, comprising hyphae that are mostly narrow, 3.5–4.5 μm wide (x- = 3.8 μm, n= 20), brownish, slightly constricted at the septa, dense, radiating outward, anastomosing at the tips with cells of the hyphal network. ***Sexual morph***: *Ascomata* 130–180 μm high, 200–250 μm diam. (x- = 155 × 220 µm, n = 10) in diameter, superficial, solitary, pale brown, globose to subglobose, coriaceous, somewhat flattened when dry, covered by a mycelial pellicle, with a circumferential space filled with sparse mycelium around the mature ascomata. *Peridium* 18–25 μm wide (x- = 23.5 μm, n= 20), light brown, with compressed, hyaline, inner cells of *textura angularis* and light brown outer cells of *textura angularis*. *Asci* (62–)70–90 × 30–60 μm (x- = 81 × 44 µm, n = 20), 8-spored, bitunicate, broadly obovoid, short pedicellate, apically rounded, with well-developed ocular chamber. *Ascospores* 30–45(–47) × 8–16 μm (x- = 36 × 12 µm, n = 30), crowded or overlapping, irregularly triseriate, hyaline, oblong to ellipsoid, muriform, with 7 transversal septa and 6 longitudinal septa, slightly constricted at the septa, smooth-walled, surrounded by a mucilaginous sheath. ***Asexual morph***: Not observed.

##### Culture characteristics.

Colonies on PDA reaching 3 mm diameter after 2 weeks at 25–30 °C, slow growing, spreading, with folded, velvety, wavy margin, consist of dark mycelium, colony color from above: olivaceous green; colony color from below: dark brown to black, not producing pigments in PDA.

##### Material examined.

Taiwan, Chiayi, Fanlu Township area, Dahu forest, decaying leaves of *Ficus ampelas* Burm.f (Moraceae), 20 June 2018, D.S. Tennakoon, H10 (MFLU19-0823), living culture (MFLUCC19-0252).

##### Notes.

In this study, a sample of *Ceramothyrium longivolcaniforme* was collected from dead leaves of *Ficus ampelas* (Moraceae) in Taiwan. The new collection shares a close phylogenetic relationship with *Ceramothyrium longivolcaniforme* (MFLU16-1306) (Figure 1). The morphology of our collection (MFLUCC19-0252) fits with the type material of *Ceramothyrium longivolcaniforme* (MFLU16-1306) in having elongate mycelial pellicle, broadly obovoid, short pedicellate asci and hyaline, oblong to ellipsoid, muriform ascospores with a mucilaginous sheath (Zeng et al. 2016). However, the ascospores are slightly larger (30–45 × 8–16 μm) than MFLU16-1306 (28–37 × 7–13 μm) (Table 2). *Ceramothyrium longivolcaniforme* has been previously reported from Thailand on unidentified sp. (not *F. ampelas*) and thus, we provide the new host record of *Ceramothyrium longivolcaniforme* on *Ficus ampelas* (Moraceae). Remarkably, this is the first *Ceramothyrium* species collected from Taiwan.

**Figure 2.**
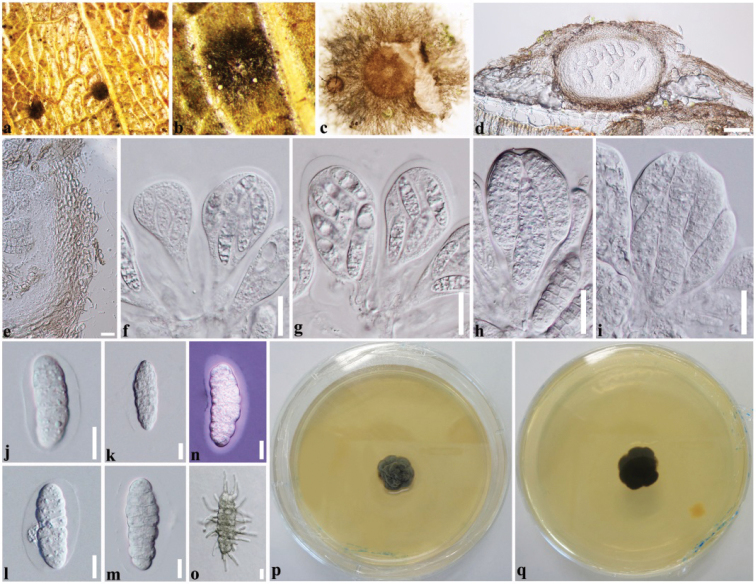
*Ceramothyrium longivolcaniforme* (MFLU19-0823, new host record). **a, b** Appearance of colony (black spots) on host leaf **c** mycelial pellicle **d** vertical section through ascoma **e** section of peridium **f–i** asci **j–m** ascospores **n** ascospore stained in Indian ink showing mucilaginous sheath **o** germinating ascospore **p, q** colony from above and below. Scale bars: 50 µm (**d**), 10 µm (**e**), 20 µm (**f–i**), 10 µm (**j–o**).

**Table 2.** Comparison of ascospore characters among species of *Ceramothyrium*.

Species	Numbers of septa	Host /Locality	Size (μm)	References
*C. anacardii*	3	–	33–50 × 7–9.5	Batista and Maia (1956)
*C. aurantii*	3–6	–	18.9–27 × 5.4–8	Batista and Maia (1956)
*C. biseptatum*	2	*Macaranga tanarius*/ Philippines	14–16 × 4.5–5.5	Batista and Ciferri (1962)
*C. boedijnii*	3	*Theobroma cacao*/ Papua New Guinea	15–20 × 5–7	Batista and Ciferri (1962)
*C. calycanthi*	6–10	*Calycanthus* sp./ Georgia	24.5–37 × 6.5–9.5	Batista and Ciferri (1962)
*C. carniolicum*	3	*Pyrola rotundifolia*/ Sweden	18–20 × 4–5.5	Eriksson (1992)
*C. cinereum*	7	–	35–42 × 7–9	Batista and Maia (1956)
*C. citricola*	3–4	*Citrus aurantium*/ Brazil	14–30 × 2.5–11	Mendes et al. (1998)
*C. coffeanum*	3	*Coffea robusta*/ New Guinea	12–16 × 4–6	Batista and Ciferri (1962)
*C. cordiae*	3	*Cordia rufescens*/ Brazil	10–13.5 × 4–5.4	Eriksson (1992)
*C. europaeum*	3	*Pogonophora schomburgkiana*/ Brazil	16–20 × 4–5.5	Eriksson (1992)
*C. globosum*	6–9 transversal	–	50–58 × 5–6	Batista and Maia (1956)
*C. griseolum*	4–6	*Aleurites moluccana*/ Brazil	19–25 × 4–5	Eriksson (1992)
*C. gustaviae*	3–5	*Gustavia augusta*/ Brazil	22–25 × 3.7–5	Eriksson (1992)
*C. gymnopogonis*	2	*Alyxia scandens*/ Samoa	15 × 5	Dingley et al. (1981)
*C. jambosae*	–	*Eugenia malaccensis*/ Brazil	–	Eriksson 1992
*C. linnaeae*	3–4	*Lycopodium annotinum*/ Sweden	12–18 × 3–5	Constantinescu et al. 1989
*C. longivolcaniforme* (MFLU 16-1306)	7 transversal	Unidentified/ Thailand	28–37 × 7–13	Zeng et al. (2016)
	6 longitudinal			
***C. longivolcaniforme* (MFLU 19-0823)**	**7 transversal**	***Ficus ampelas* / Taiwan**	**30–45 × 8–16**	**This study (New host record)**
	**6 longitudinal**			
*C. lycopodii*	7	*Lycopodium annotinum*/ Sweden	45 × 4	Constantinescu et al. (1989)
*C. martinii*	5–7	–	20–27 × 7–9	Barr (1993)
*C. moravicum*	2–3	–	10–14 × 3–5	Petrak (1961)
*C. paiveae*	1–4	*Paivaea langsdorffii*/ Brazil	12.5–22 × 3.7–6	Mendes et al. (1998)
*C. paraense*	3–7	*Anacardium* sp./ Brazil	20–30 × 3.5–4	Mendes et al. 1998
*C. parenchymaticum*	5–7	*Didymopanax morototoni*/ Cuba	30–40 × 8–10	Batista and Ciferri 1962
*C. peltatum*	6–9	–	28–32 × 4.5–6.5	Batista and Maia (1956)
*C. philodendri*	1–7	*Philodendron imbe*/ Brazil	17.5–32.5 × 5–7.5	Mendes et al. (1998)
*C. thailandicum*	7–9 transversal	*Lagerstroemia* sp./ Thailand	24.7–35.5 × 5.7–8.7	Chomnunti et al. (2012)

#### Longihyalospora

Taxon classificationAnimaliaChaetothyrialesChaetothyriaceae

Tennakoon, C.H Kuo & K. D Hyde gen. nov.

BA6AF1D7-4875-50A6-A448-7A5329BE7CFB

Index Fungorum number: IF 556715

Facesoffungi number: FoF06136

##### Etymology.

Referring to the long, hyaline ascospores.

##### Description.

*Epiphytic* on the upper surface decaying leaves, appearing as small black dots. Covering the upper leaf surface with dark mycelium without penetrating host tissues. *Mycelial pellicle* elongate, subiculum-like, comprising hyphae that are mostly narrow, dense, dark brown. *Mycelial setae* broad, dark brown, scattered, discrete, arranged as a ring around the pellicle, unbranched, formed on dense, dark hyphae. ***Sexual morph***: *Ascomata* superficial, solitary, dark brown to black, globose to subglobose, coriaceous, uni-locular, somewhat flattened when dry, covered by a mycelial pellicle. *Peridium* pale brown to brown, with compressed, hyaline, inner cells of *textura angularis* and dark brown outer cells of *textura angularis*, fusing and indistinguishable from the host tissues. *Asci* 8-spored, bitunicate, broadly obovoid, slightly stalked, apically rounded, with a well-developed ocular chamber. *Ascospores* overlapping, irregularly triseriate, hyaline, fusiform, elongated, multi-septate, slightly constricted at the septa, tapering to the ends, smooth-walled, surrounded by a thin mucilaginous sheath. ***Asexual morph***: Not observed.

##### Type species.

*Longihyalospora ampeli* Tennakoon, C.H Kuo & K. D Hyde.

#### Longihyalospora ampeli

Taxon classificationAnimaliaChaetothyrialesChaetothyriaceae

Tennakoon, C.H Kuo & K.D. Hyde sp. nov.

F066F419-72D8-5847-AB64-0D4E5AC2B474

Index Fungorum number: IF 556716

Facesoffungi number: FoF06137

Figure 3

##### Etymology.

Species name based on the host *Ficus ampelas*, from which it was collected.

##### Holotype.

MFLU 19-0824

##### Description.

*Epiphytic* on the upper surface decaying leaves, appearing as small black dots. Covering the upper leaf surface with dark mycelium without penetrating host tissues. *Mycelial* pellicle (190–) 200–250 (–258) µm diam., elongate, subiculum-like, comprising hyphae that are mostly narrow, 1–2 μm wide (x- = 1.5 μm, n= 20), dense, dark brown. *Mycelial setae* (197–) 200–225 (–231) µm long, at base 10–12 µm wide, at apex 2–3 µm wide, dark brown, scattered, discrete, arranged as a ring around the pellicle, unbranched, formed on dense, dark hyphae. ***Sexual morph***: *Ascomata* 55–90 μm high, 150–200 μm diam. (x- = 76 × 168 µm, n = 10) in diameter, superficial, solitary, dark brown to black, globose to subglobose, coriaceous, uni-locular, somewhat flattened when dry, covered by a mycelial pellicle. *Peridium* 18–25 μm wide (x- = 23.5 μm, n= 20), pale brown to brown, with compressed, hyaline, inner cells of *textura angularis* and dark brown outer cells of *textura angularis*. *Asci* (82–) 90–115 (–120) × 52–62 μm (x- = 106 × 57 µm, n = 20), 8-spored, bitunicate, broadly obovoid, slightly stalked, apically rounded, with well-developed ocular chamber. *Ascospores* (74–) 76–98(–105) × 10–12 μm (x- =84 × 10.8 µm, n = 30), overlapping, irregularly triseriate hyaline, elongate fusiform, (6–) 8–11 (–12) septa, slightly constricted at the middle septum, tapering to the ends, smooth-walled, surrounded by a 3.5–5 µm wide mucilaginous sheath. ***Asexual morph***: Not observed.

##### Material examined.

Taiwan, Chiayi, Fanlu Township area, Dahu forest, decaying leaves of *Ficus ampelas* (Moraceae), 20 June 2018, D.S. Tennakoon, H50B1 (MFLU 19-0824, **holotype**), H50B2 (MFLU19-0825, **isotype**).

##### Notes.

*Longihyalospora* is described herein as a new monotypic genus in Chaetothyriaceae. *Longihyalospora* differs from other genera in Chaetothyriaceae by a combination of a dark mycelium covering the upper leaf surface, an elongate mycelial pellicle, ring of setae around the pellicle, pale brown to brown peridium with hyaline inner layers, broadly obovoid, short pedicellate asci and hyaline, elongate fusiform and 8–11-septate ascospores, with tapering ends and a thin mucilaginous sheath. In our phylogenetic analyses, *Longihyalospora ampeli* species constitutes a strongly supported sub clade, which is nested independently from other genera in Chaetothyriaceae (Figure 1).

**Figure 3.**
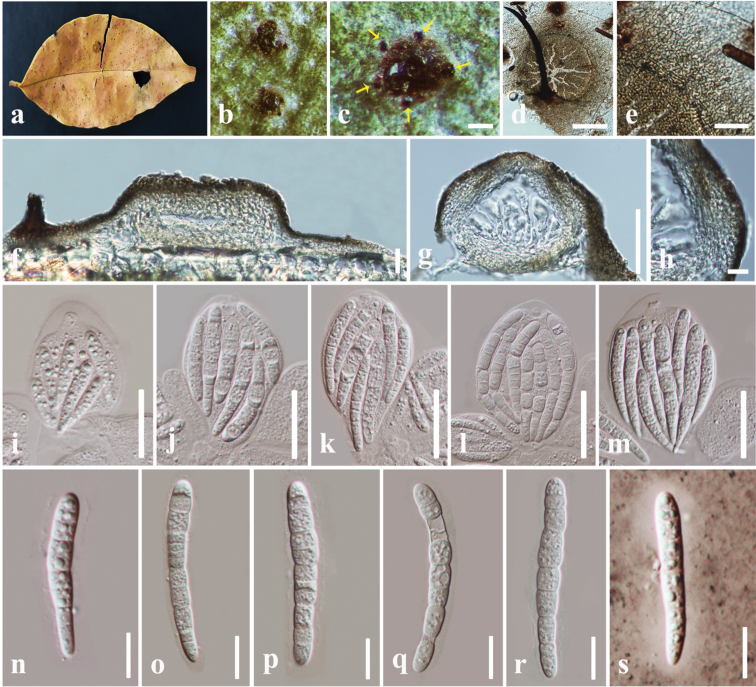
*Longihyalospora ampeli* (MFLU 19-0824, holotype). **a** Host leaf **b** appearance of colony (black spots) on leaf **c** ring of setae around the pellicle **d** mycelial pellicle with setae **e** mycelial pellicle cells **f, g** vertical section through ascoma **h** section of peridium **i–m** asci **n–r** ascospores **s** ascospore stained in Indian ink showing a mucilaginous sheath. Scale bars: 100 µm (**c**), 75 µm (**d**), 20 µm (**e, f**), 50 µm (**g**), 10 µm (**h**), 50 µm (**i–m**), 20 µm (**n–s**).

#### Longihyalospora vermisporum

Taxon classificationAnimaliaChaetothyrialesChaetothyriaceae

(Hansf.) Tennakoon, C.H. Kuo & K.D. Hyde comb. nov.

C47738F9-D32D-5CD5-996C-89B275C7FD0B

Index Fungorum number: IF 556717

Facesoffungi number: FoF01679

≡ Chaetothyrium vermisporum Hansf., Mycol. Pap. 15: 151 (1946). *Morphological description*: See Hansford (1946), Hofmann and Piepenbring (2006).

##### Recorded hosts.

*Canthium* sp. (Rubiaceae) Hansford no. 1327; *Hugonia platysepalae* (Linaceae) Hansford no. 1384; *Ventilago africana* (Rhamnaceae), Hansford no. 2930 (Hansford, 1946).

##### Known distribution.

Uganda (Hansford, 1946), Panama (Hofmann and Piepenbring 2006).

##### Notes.

*Chaetothyrium vermisporum* was introduced by Hansford (1946) which was collected from Uganda based on morphological characteristics. Subsequently, it has been collected from Panama by Hofmann and Piepenbring (2006). After in-depth morphological investigations, we found that *Chaetothyrium vermisporum* shares some similar morphology with *Longihyalospora ampeli* by having mycelial pellicle with ring of setae, pale brown to brown peridium and hyaline, fusiform, elongated and multi-septate ascospores (Hansford (1946). However, *Chaetothyrium vermisporum* can be distinguished from *Longihyalospora ampeli* by having hyaline surface mycelium, smaller asci (60 × 30 µm) and ascospores (35–50 × 5–6 µm) without a mucilaginous sheath, whereas *Longihyalospora ampeli* has dark brown mycelium, larger asci (90–115 × 52–62 µm) and ascospores (76–98 × 10–12 μm) with mucilaginous sheath. Therefore, we synonymized *Chaetothyrium vermisporum* under *Longihyalospora* based on high morphological similarities. Fresh collections with molecular data are needed to clarify the phylogenetic affinity of *Longihyalospora vermisporum*.

Additionally, we compared our collection with *Chaetothyrium guaraniticum* Speg. (type species of *Chaetothyrium*). *Longihyalospora ampeli* can be distinguished from *Chaetothyrium guaraniticum* by many morphological characters, viz. *C. guaraniticum* has 1-septate shorter ascospores (10–14 × 4–5 µm) and lacks a mucilaginous sheath (Spegazzini 1888), whereas *L. ampeli* has multi-septate (8–11), longer (84 × 10.8 µm) ascospores with a mucilaginous sheath. Further collections are needed to resolve the phylogenetic position and relationships between members of *Chaetothyrium* and *Longihyalospora* species.

## Discussion

Sooty molds are an interesting group of fungi in tropical and temperate regions in worldwide (Chomnunti et al. 2014; Hongsanan et al. 2015; Farr and Rossman 2019; Kwon et al. 2019). Their morphology has been well-studied but their phylogenetic relationships are poorly understood due to the difficulty of obtaining good-quality DNA samples (Chomnunti et al. 2011, 2014; Zeng et al. 2016; Zeng et al. 2019). Currently, seven sooty mold forming families have been reported, viz. Antennulariellaceae Woron., Capnodiaceae Höhn., Euantennariaceae S. Hughes & Corlett ex S. Hughes, Metacapnodiaceae S. Hughes & Corlett (Dothideomycetes) and Chaetothyriaceae Hansf. ex M.E. Barr, Coccodiniaceae Höhn. ex O.E. Erikss., and Trichomeriaceae Chomnunti & K.D. Hyde (Eurotiomycetes) (Reynolds 1998; Winka et al. 1998; Hughes and Seifert 2012; Hyde et al. 2013; Chomnunti et al. 2014; Hongsanan et al. 2016).

Chaetothyriaceae species are widespread in tropical and temperate regions (Hofmann and Piepenbring 2006; Chomnunti et al. 2011, 2014; Hongsanan et al. 2015; Zeng et al. 2016; Maharachchikumbura et al. 2018; Yang et al. 2018; Farr and Rossman 2019). Wijayawardene et al. (2018) accepted 16 genera in Chaetothyriaceae, but currently only seven genera (*Aphanophora*, *Camptophora*, *Ceramothyrium*, *Chaetothyrium*, *Cyphellophoriella*, *Phaeosaccardinula* and *Vonarxia*) have DNA sequence data. The main morphological differences of Chaetothyriaceae genera are mentioned in Table 3.

**Table 3.** Synopsis of sexual morphs of Chaetothyriaceae genera discussed in this study.

Genus name	Ascomata or mycelium setose/glabrous	Asci		Ascospores				References
		Shape	Number of spores/ascus	Shape	Color	Septation	Sheath	
*Actinocymbe* Höhn.	Glabrous	straight to sickle shape	8	club shaped	hyaline to light brown	9		Verma and Kamal (1987)
*Beelia* F. Stevens & R.W. Ryan	Glabrous	broadly ellipsoidal	8	cylindrical	hyaline	5	yes	Li et al. (2011)
*Camptophora* Réblová & Unter.	Glabrous	long-ellipsoid to obovoid	8	obovoid to pyriform	hyaline	1–3 or muriform	no	Yang et al. (2018)
*Ceramothyrium* Bat. & H. Maia	Glabrous	clavate or pyriform	8	oblong to ellipsoid or cylindrical clavate	hyaline	3–10 or muriform	yes	Zeng et al. (2016), Chomnunti et al. (2012)
*Ceratocarpia* Rolland	Glabrous	clavate to broadly clavate	8	ellipsoid to fusiform	light brown	muriform	no	Tian et al. (2014)
*Chaetothyrium* Speg.	Setose	broadly ovoid or oblong	8	oblong to ellipsoidal or obovoid	hyaline	4–7 or muriform	no	Chomnunti et al. (2012), Liu et al. (2015)
*Chaetothyriomyces* Pereira-Carv et al.	Glabrous	broadly clavate	16	elliptical	hyaline	1	no	Pereira et al. (2009)
*Euceramia* Bat. & Cif.	Glabrous	ellipsoid to pyriform	8	clavate-fusoid	hyaline	4–5	no	Batista and Ciferri (1962)
***Longihyalospora* Tennakoon, C.H. Kuo & K.D. Hyde**	**Setose**	**broadly obovoid**	**8**	**fusiform and elongated**	**hyaline**	**8–11**	**yes**	**This study**
*Microcallis* Syd.	Glabrous	clavate	8	oblong to clavate	hyaline	1	no	Sydow (1926), Chomnunti et al. (2011)
*Phaeosaccardinula* Henn.	Glabrous	obovoid to oval	4–6	oblongellipsoid to reniform	hyaline or pale brown	muriform	yes	Yang et al. (2014), Maharachchikumbura et al. (2018)
*Treubiomyces* Höhn.	setose	clavate	8	oblong to clavate	hyaline	muriform	no	Höhnel (1909), Pohlad (1989)
*Yatesula* Syd. & P. Syd.	Glabrous	clavate	4–8	oblong to clavate	brownish yellow	3–4 or muriform	no	Ellis and Everhart, (1893), Sydow and Sydow (1917)

Batista and Maia (1956) established the genus *Ceramothyrium* and designated *Ceramothyrium paiveae* Bat. & H. Maia as the type species, which has been collected from Brazil. *Ceramothyrium* species are characterized by a mycelial pellicle that covers the ascomata with a circumferential space around the maturing ascomata, lack of setae and hyaline, transversely pluriseptate ascospores (Batista and Maia 1956; Chomnunti et al. 2012; Tsurumi et al. 2018). Most *Ceramothyrium* species have been collected from terrestrial habitats and their asexual morph has been recorded as *Stanhughesia* Constant. (Chomnunti et al. 2012; Réblová et al. 2013; Wijayawardene et al. 2017; Tsurumi et al. 2018). *Ceramothyrium* species seem to have a diverse distribution since they have been recorded from both temperate and tropical countries (i.e. Brazil, Canada, Georgia, Indonesia, Thailand, Panama, Philippines, South Africa, Sweden, Vietnam) (Hofmann and Piepenbring 2006; Chomnunti et al. 2012; Crous et al. 2012; Zeng et al. 2016; Tsurumi et al. 2018; Farr and Rossman 2019). Host-specificity of the taxa in this group has not yet been proven, since they have been recorded from various plant families (i.e. Arecaceae, Anacardiaceae, Ericaceae, Lycopodiaceae, Lythraceae, Melastomataceae, Podocarpaceae, Rubiaceae) (Batista and Maia 1956; Chomnunti et al. 2012; Hongsanan et al. 2015; Farr and Rossman 2019). Combined phylogenetic analyses with a larger taxon sampling provide a better resolution of interspecific relationships of *Ceramothyrium* within Chaetothyriaceae (Chomnunti et al. 2014; Zeng et al. 2016; Maharachchikumbura et al. 2018; Yang et al. 2018).

Recent studies have revealed that *Ceramothyrium* is a species rich genus. For instance, in the last few years, numerous *Ceramothyrium* species have been described. *Ceramothyrium longivolcaniforme*, *C. menglunense* were introduced by Zeng et al. (2016) and Hyde et al. (2016) respectively. Yen et al. (2018) introduced three *Ceramothyrium* species, viz. *C. aquaticum*, *C. phuquocense* and *C. exiguum*. Currently, there are 41 *Ceramothyrium* epithets in Index Fungorum (2019).

Most previous Chaetothyriaceae studies have been based on brief descriptions with line drawings and without DNA sequence data (i.e. *Actinocymbe*, *Beelia*, *Ceratocarpia*, *Chaetothyriomyces*, *Euceramia*, *Microcallis*, *Stanhughesia*, *Treubiomyces* and *Yatesul*a). Therefore, it is essential to focus on DNA sequence data to clarify the phylogenetic affinity of above genera in Chaetothyriaceae in future studies. Thus, it is necessary to collect more fungi similar to Chaetothyriaceae in different geographic regions and hosts, isolate them into cultures, describe their morphology, analyze their DNA sequences and investigate their phylogenetic relationships for a better identification and classification.

## Supplementary Material

XML Treatment for Ceramothyrium longivolcaniforme

XML Treatment for Longihyalospora

XML Treatment for Longihyalospora ampeli

XML Treatment for Longihyalospora vermisporum
